# Nomogram based on circulating lymphocyte subsets for predicting radiation pneumonia in esophageal squamous cell carcinoma

**DOI:** 10.3389/fimmu.2022.938795

**Published:** 2022-08-29

**Authors:** Xiao-zhen Zhang, Su-ping Tao, Shi-xiong Liang, Shu-bin Chen, Fu-shuang Liu, Wei Jiang, Mao-jian Chen

**Affiliations:** ^1^ Department of Medical Oncology, Sun Yat-sen University Cancer Center, State Key Laboratory of Oncology in South China, Collaborative Innovation Center for Cancer Medicine, Guangzhou, China; ^2^ Department of Radiation Oncology, Guangxi Medical University Cancer Hospital, Nanning, China; ^3^ Department of Respiratory Oncology, Guangxi Medical University Cancer Hospital, Nanning, China

**Keywords:** esophageal squamous cell carcinomas, circulating lymphocyte subsets, nomogram, radioactive pneumonia, CD8+T cells, immune response

## Abstract

**Purpose:**

Currently, the relationship between radiation pneumonia (RP) and circulating immune cell in patients with esophageal squamous cell carcinoma (ESCC) remains unclear. This study aimed to explore the relationship between RP and circulating lymphocyte subsets in patients with ESCC receiving chemoradiotherapy (CRT), and develop a nomogram model to predict RP. Since we should implement clinical intervention to ≥ grade 2 RP, a nomogram model for ≥ grade 2 RP was also established to provide an early warning.

**Patients and methods:**

This study retrospectively included 121 patients with ESCC receiving CRT from Guangxi Medical University Cancer Hospital from 2013 to 2021. Independent factors associated with occurrence of RP and ≥ grade 2 RP were identified by univariate and multivariate logistic regression analysis in the training cohort, and incorporated into nomograms. The predictive accuracy and discrimination of the model was assessed using Concordance Index (C-index), calibration curve and decision curve analysis (DCA). And each model was internally validated. Additionally, to verify the optimized predictive performance of the nomograms, the area under the ROC curve (AUC) of each nomogram was compared to that of single independent risk factors, lung V10 and lung V20, respectively. Moreover, each model was further evaluated for risk stratification to identify populations at high risk of RP and ≥ grade 2 RP.

**Results:**

Multivariate analysis suggested that TNM stage, post-RT percentage of CD8+ T cell, and lung V15 were independent predictive factors of RP. Besides, pre- and post-RT percentage of CD8+ T cell, and V15 were independent factors of ≥ grade 2 RP. The C-indexes of RP and ≥ grade 2 RP nomograms were 0.809 (95% CI: 0.715-0.903) and 0.787 (95% CI: 0.685-0.889) in the training cohort, respectively. And the C-indexes of RP and ≥ grade 2 RP nomograms were 0.718 (95% CI: 0.544-0.892) and 0.621 (95% CI: 0.404-0.837) in the validation cohort, respectively. The calibration curves showed that the predicted values of model agreed well with actual observations. Moreover, DCA results indicated the applicability and accuracy of the models to predict RP and ≥ grade 2 RP. After stratification, the incidence of the high-risk group was significantly higher than that of the low-risk group with respect to either RP or ≥ grade 2 RP.

**Conclusion:**

TNM stage, post-RT percentage of CD8+ T cell, and lung V15 were the independent predictors of RP toxicity. Pre- and post-RT percentage of CD8+ T cell, and lung V15 were the independent factors of ≥ grade 2 RP toxicity. The nomograms based on circulating lymphocyte subsets can robustly predict RP and ≥ grade 2 RP, guiding clinicians in risk stratification and early intervention.

## Introduction

Esophageal cancer is the seventh most common malignancy worldwide, and the sixth leading cause of cancer-related death, causing an estimated 508,000 deaths in 2018 (1, 2). Esophageal cancer is mainly classified as esophageal adenocarcinoma (EAC) and esophageal squamous cell carcinoma (ESCC) (3, 4). ESCC has predominantly occurred in Eastern Asia and some regions of Africa. Unfortunately, ESCC is often diagnosed as locally advanced stage, with only 18% overall 5-year survival (5). The standard treatment for locally advanced ESCC is chemoradiotherapy (CRT) (6, 7). According to RTOG 85-01 study, CRT can significantly improve survival, with a 5-year overall survival of 26% (8).

Radiation pneumonia (RP) is a possible complication of CRT for ESCC. RP may cause dyspnea and chronic pulmonary fibrosis (9, 10). Then, RP is one of the major reasons that limits the dose of radiation therapy (RT) and impairs the efficacy of RT (11, 12). Besides, RP may compromise quality of life post-RT in patients receiving RT. Serious RP even results in treatment-related death (9, 13). Accordingly, early detection and intervention are crucial for the management of RP, and a robust biomarker for the prediction of RP is of urgent needed.

Previous studies have focused on clinical and dose parameters for the prediction of RP, for example, smoking history, lung V10 and lung V20 (14–17). However, these factors showed limited efficiency for individualized RP prediction. As is well known, RT can induce an immune response through the release of pro-inflammatory cytokines and the regulation of immune cells (18, 19), while RP has a closed connection with inflammatory cells infiltrated (20, 21). Specially, lymphocyte was show to closely related to the risk of RP, with the recruitment of lymphocyte in the process of RP (22, 23). Nevertheless, it is reasonable to infer that lymphocyte subsets with differentiated activity involving in inflammation maybe the potential predictor of RP. Lymphocytes are mainly divided into T cell, B cell, and natural killer cell (24). And T cells are further divided into helper T cell (CD4+) and suppressor/cytotoxic T cell (CD8+) (24, 25). Given that the value of circulating lymphocyte subsets in the prediction of RP remains unclear, we sought to explore the relationship between circulating lymphocyte subsets and RP in ESCC, and develop a nomogram model to predict RP. Since we should implement clinical intervention to ≥ grade 2 RP, a nomogram model for ≥ grade 2 RP was also established to provide an early warning.

## Patients and methods

We retrospectively screened a total of 258 patients diagnosed with ESCC at Guangxi Medical University Cancer Hospital from 2013 to 2021. Among them, 121 patients receiving definitive intensity‐modulated radiation therapy (IMRT) and concurrent chemotherapy were enrolled. All included patients had CT scan post-RT at about two-months intervals for the evaluation of treatment efficacy and RP. In addition, circulating lymphocyte subsets detections of included patients at baseline and post-RT within six weeks were required. The exclusion criteria were as follow: (1) related diseases affecting cellular immunity and humoral immunity, such as acquired immune deficiency syndrome, rheumatoid arthritis, systemic lupus erythematosus, hypersensitivity disease, and immunodeficiency diseases; (2) synchronous malignancy; (3) severe lung infection; (4) interstitial pneumonia at baseline; (5) incomplete data. Since the retrospective nature of the study, informed consent was waived, and patient information was confidential and anonymized. The Ethics Committee of Guangxi Medical University Cancer Hospital approved the study protocol.

### Radiotherapy and chemotherapy

All patients were treated with IMRT using a 6-MV linear accelerator (Elekta Synergy, Stockholm, Sweden). The target area and organs at risk (OARs) referred to the Radiotherapy and Oncology Group (RTOG) guidelines. The gross tumor volume (GTV) and metastatic lymph nodes (GTVnd) were defined as follows: the horizontal extension of GTV/GTVnd by 0.5 cm, the craniocaudal margin extension of GTV by 3-5 cm, and the craniocaudal margin extension of GTVnd by 0.5 cm. The planning target volume (PTV) was determined by adding a 0.5 cm margin to the clinical target volume (CTV), and 95% isodose curve covers 100% target volume. The prescribed dose of planning gross tumor volume (PGTV) was 50-69.96 Gy/25-33 f, and the fractional dose was 1.8-2.2 Gy. The dose of planning clinical target volume (PCTV) ranged from 50.4 to 60.06 Gy, with 1.8-1.82 Gy for per fraction. Strictly limited dose range of normal tissue include: heart V40 < 30% and heart V30 < 40%; lung V20 < 30% and lung V30 < 20%; spinal cord ≤ 45Gy. Dose-volume histograms (DVH) were used to assess the dose distribution of treatment plan for each patient before treatment. Patients received radiotherapy with five fractions per week and one fraction per day.

Concurrent chemotherapy was administrated at 3-week intervals for up to 6 cycles. The chemotherapy regimens were platinum-based (cisplatin, nedaplatin and carboplatin) including: platinum + paclitaxel, platinum + 5-fluorouracil, platinum + etoposide, and platinum + docetaxel).

### Flow cytometry analysis

Flow cytometry assay was used to detect the percentage of the circulating lymphocyte subsets from the peripheral blood including CD4+ T cell (CD4+), CD8+ T cell (CD8+), natural killer cell (CD3- CD16+ CD56+) and B cell (cCD19+) at baseline and post-RT within six weeks. Experimental protocols of the cellular immunology related indicators were referred to a previously described method (26).

### Follow-up and statistical analysis

All included patients underwent contrast-enhanced CT scan for efficacy evaluation and RP detection at intervals of about two months during the first two years post-RT. RP was graded according to the National Cancer Institute Common Toxicity Criteria for Adverse Events version 4.0 (CTCAE v4.0).

The included patients were divided into training cohorts and validation cohorts in a ratio of 7:3. Student’s t-test or nonparametric test was used to compare the continuous variables, while the Pearson’s chi-square test or Fisher’s exact was used to compare the categorical variables. We included age, sex, smoking history, TNM stage, tumor location, chemotherapy regimens, the percentage of pre- and post-RT circulating lymphocyte subsets (CD4+ T cell, CD8+ T cell, natural killer cell and B cell), PGTV and irradiated lung volume at different dose levels (V5, V10, V20, V25 and V30) for analysis. The logistic regression model was used for univariate and multivariate analysis to identify the risk factors associated with RP and ≥ grade 2 RP. Independent variables (*p*<0.05) in the multivariate analysis were incorporated into the nomogram models to predict RP and ≥ grade 2 RP, respectively. In the training cohort, Harrell’s C-index (C-index) and the receiver operating characteristic (ROC) analysis were used to estimate the discriminant performance of the nomogram. The calibration curve constructed by bootstrap verification with 1000 resamples was used to compare the predictive probability of RP with the observed RP. The effectiveness of the nomogram was further evaluated in the validation cohort. Decision curve analysis (DCA) was used to assess whether patients could benefit from intervention based on the nomogram model. The best cutoff value of the model risk score was determined using the ROC analysis. And all patients in training cohort were assigned to low- and high-risk groups according to the cutoff value. The *p* value < 0.05 was considered statistically significant. All statistical analyses were carried out using SPSS (version 26.0) and R (version 4.1.1).

## Results

### Patient characteristics

A total of 121 patients were included, and randomly assigned to the training cohort (n=85) and the validation cohort (n=36), respectively. The characteristics of patient and tumor at baseline, and data on treatment, and pre- and post-RT circulating lymphocyte subsets were summarized in **Table 1**.

**Table 1:** Characteristics of patient, tumor and treatment.

Variables	Overall (n = 121)	Training cohort (n = 85)	Validation cohort (n = 36)	*p*
Age(year)				0.056
<56	53 (43.8%)	42 (49.4%)	11 (30.6%)	
≥56	68 (56.2%)	43 (50.6%)	25 (69.4%)	
Sex				0.482
Female	10 (8.3%)	6 (7.1%)	4 (11.1%)	
Male	111 (91.7%)	79 (92.9%)	32 (88.9%)	
Smoking history				0.495
No	39 (32.2%)	29 (34.1%)	10 (27.8%)	
Yes	82 (67.8%)	56 (65.9%)	26 (72.2%)	
TNM stage				0.493
II+III	89 (73.6%)	61 (71.8%)	28 (77.8%)	
IV	32 (26.4%)	24 (28.2%)	8 (22.2%)	
Tumor location				0.752
cervical/upper	27 (22.3%)	20 (23.5%)	7 (19.4%)	
middle	45 (37.2%)	33 (38.8%)	12 (33.3%)	
lower	18 (14.9%)	11 (12.9%)	7 (19.4%)	
multifocal	31 (25.6%)	21 (24.7%)	10 (27.8%)	
Chemotherapy regimens				0.944
platinum + paclitaxel	70 (57.9%)	49 (57.6%)	21 (58.3%)	
platinum + non-paclitaxel	51 (42.1%)	36 (42.4%)	15 (41.7%)	
Percentage of pre-RT circulating lymphocyte subsets				
pre -RT CD4+ T cell (%, median [IQR])	41.1 [34.45, 47.55]	40.2 [34.45, 47]	41.3 [34.25, 48.45]	0.632
pre- RT CD8+ T cell (%, median [IQR])	20.2 [16.4, 24.45]	20 [16.3, 23.17]	21.7 [17.13, 29.25]	0.035
pre- RT NK cell (%, median [IQR])	12.6 [8.35, 18.2]	12.6 [9.15, 18.65]	10.65 [6.28, 17.43]	0.216
pre- RT B cell (%, median [IQR])	9.8 [6.7, 14]	9.88 [6.45, 15]	9.59 [7.05, 13.23]	0.943
Percentage of post-RT circulating lymphocyte subsets				
post-RT CD4+ T cell (%, median [IQR])	24.1 [17.2, 30.75]	25.1 [18.65, 30.9]	19.65 [15.33, 26.6]	0.015
post-RT CD8+ T cell (%, median [IQR])	35.9 [27.15, 43.65]	34.1 [25.2, 39.61]	43.15 [35.38, 57.78]	<0.0001
post-RT NK cell (%, median [IQR])	12.1 [8.8, 16.25]	12.6 [10.2, 16.8]	9.85 [6.53, 14.33]	0.051
post-RT B cell (%, median [IQR])	3.88 [1.64, 8.1]	3.5 [1.75, 8.2]	4.05 [1.35, 7.95]	0.892
PGTV (Gy) (median [IQR])	60 [60, 63]	62 [60, 63]	60 [60, 63]	0.148
Lung V5 (%, median [IQR])	68.38 [57.54, 80.02]	68.38 [58.31, 79.69]	68.06 [56.23, 88.31]	0.943
Lung V10 (%, median [IQR])	48 [42.57, 57.32]	48 [43.17, 56]	46.79 [41.06, 61.22]	0.616
Lung V15 (%, median [IQR])	36.61 [33.38, 41.31]	37 [33.66, 41.30]	36.02 [31.31, 41.76]	0.586
Lung V20 (%, median [IQR])	27.94 [25, 29.41]	28 [25, 29.71]	27.9 [25.21, 29.3]	0.523
Lung V25 (%, median [IQR])	20.95 [18.27, 23.04]	20.95 [18.84, 23.46]	20.81 [18.06, 22.89]	0.489
Lung V30 (%, median [IQR])	16 [13.85, 18.7]	16 [14.09, 19]	16.30 [13.18, 17.14]	0.395

Platinum includes cisplatin, nedaplatin and carboplatin. Non-paclitaxel includes 5-fluorouracil, etoposide and docetaxel.

IQR, interquartile range; pre-RT, pre-radiotherapy; post-RT, post-radiotherapy; PGTV, planning gross tumor volume.

In the training cohort (n=85), we observed RP in 42 patients (49.4%), and ≥ grade 2 RP in 19 patients (22.4%); While in the validation cohort(n=36), RP in 19 patients (52.8%), and ≥ grade 2 RP in 8 (22.2%) patients.

### Determination of independent predictors for RP and ≥ grade 2 RP

Univariate analysis of logistic regression showed that TNM stage (*p*=0.022), pre-RT percentage of CD8+ T cell (*p*=0.013), post-RT percentage of CD8+ T cell (*p*=0.001), lung V10 (*p*=0.013) and lung V15 (*p*=0.007) were significantly associated with the risk of RP toxicity. And the significant factors for ≥ grade 2 RP were pre-RT percentage of CD8+ T cell (*p*=0.013), post-RT percentage of CD8+ T cell (*p*=0.023), V10 (*p*=0.012) and V15 (*p*=0.016) of lung (**Table 2**).

**Table 2:** Univariate logistic regression analysis in training cohort.

Variables	RP		RP (grade≥2)	
	OR (95% CI)	*p*	OR (95% CI)	*p*
Age	1.152 (0.492-2.699)	0.744	1.461(0.521-4.095)	0.471
Sex	0.463 (0.08-2.677)	0.39	0.254 (0.047-1.379)	0.112
Smoking history	1.635 (0.66-4.047)	0.288	3.467 (0.918-13.085)	0.067
TNM stage	0.306 (0.111-0.845)	0.022	0.883 (0.279-2.795)	0.833
Tumor location	0.914 (0.619-1.348)	0.648	1.095 (0.69-1.739)	0.7
Chemotherapy regimens	1.263 (0.534-2.991)	0.595	0.744 (0.26-2.13)	0.582
Percentage of pre-RT circulating lymphocyte subsets				
pre-RT CD4+ T cell	0.966 (0.922-1.011)	0.137	0.986 (0.936-1.039)	0.608
pre-RT CD8+ T cell	0.880 (0.796-0.973)	0.013	0.857 (0.759-0.968)	0.013
pre-RT NK cell	1.045 (0.991-1.103)	0.105	1.034 (0.976-1.094)	0.257
pre-RT B cell	1.002 (0.936-1.073)	0.955	0.929 (0.845-1.022)	0.13
Percentage of post-RT circulating lymphocyte subsets				
post-RT CD4+ T cell	0.999 (0.953-1.047)	0.965	0.998 (0.943-1.055)	0.93
post-RT CD8+ T cell	0.922 (0.878-0.967)	0.001	0.941 (0.892-0.991)	0.023
post-RT NK cell	1.064 (0.988-1.145)	0.1	1.066 (0.984-1.156)	0.119
post-RT B cell	1.013 (0.966-1.063)	0.594	0.957 (0.879-1.042)	0.312
PGTV (Gy)	0.972 (0.839-1.125)	0.701	0.883 (0.74-1.054)	0.169
Lung V5	1.031 (0.998-1.064)	0.065	1.029 (0.992-1.068)	0.126
Lung V10	1.061 (1.013-1.111)	0.013	1.062 (1.013-1.112)	0.012
Lung V15	1.12 (1.032-1.216)	0.007	1.106 (1.019-1.2)	0.016
Lung V20	1.133 (0.981-1.31)	0.09	1.179 (0.968-1.436)	0.101
Lung V25	1.024 (0.908-1.156)	0.695	1.049 (0.903-1.22)	0.529
Lung V30	1.064 (0.942-1.201)	0.317	1.085 (0.930-1.266)	0.302

OR, Odds Ratio; CI, Confidence interval; pre-RT, pre-radiotherapy; post-RT, post-radiotherapy; PGTV, planning gross tumor volume.

Multivariate logistic regression analysis was performed on the significant variables (*p*<0.05) determined by univariate analysis. First, correlational analysis was conducted to disregard the effect of multicollinearity among the variables (**Supplemental Materials**, **Table S1**). Given that spearman correlation analysis suggested significant correlation between lung V10 and lung V15, we only include lung V15 into subsequent multivariate analysis. Multivariate analysis revealed that TNM stage (OR=0.301, 95% CI: 0.095-0.954, *p*=0.041), post-RT percentage of CD8+ T cell (OR=0.923, 95% CI: 0.875-0.974, *p*=0.004) and lung V15 (OR=1.117, 95% CI: 1.018-1.224, *p*=0.019) were the independent predictors for the occurrence of RP toxicity, while pre-RT percentage of CD8+ T cell (OR=0.872, 95% CI: 0.766-0.992, *p*=0.038), post-RT percentage of CD8+ T cell (OR=0.946, 95% CI: 0.895-1, *p*=0.050), and lung V15 (OR=1.097, 95% CI: 1.008-1.195, *p*=0.032) were the independent predictors for ≥ grade 2 RP toxicity (**Table 3**).

**Table 3:** Multivariate logistic regression analysis in training cohort.

Variables	RP		RP (grade≥2)	
	OR (95% CI)	*P*	OR (95% CI)	*P*
TNM stage	0.301 (0.095-0.954)	0.041	–	–
Percentage of pre-RT CD8+T cell	0.906 (0.809-1.016)	0.09	0.872 (0.766-0.992)	0.038
Percentage of post-RT CD8+T cell	0.923 (0.875-0.974)	0.004	0.946 (0.895-1)	0.050
Lung V15	1.117 (1.018-1.224)	0.019	1.097 (1.008-1.195)	0.032

OR, Odds Ratio; CI, Confidence interval; pre-RT, pre-radiotherapy; post-RT, post-radiotherapy.

### Establishment and validation of the nomograms

**Figure 1:**
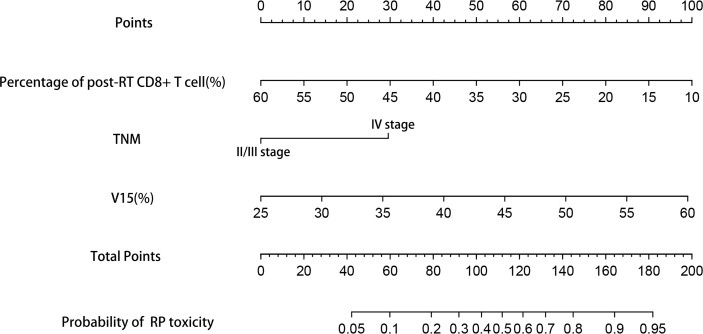
Nomogram of the probability of RP toxicity in the training cohort. pre-RT, pre-radiotherapy; post-RT, post-radiotherapy.

**Figure 2:**
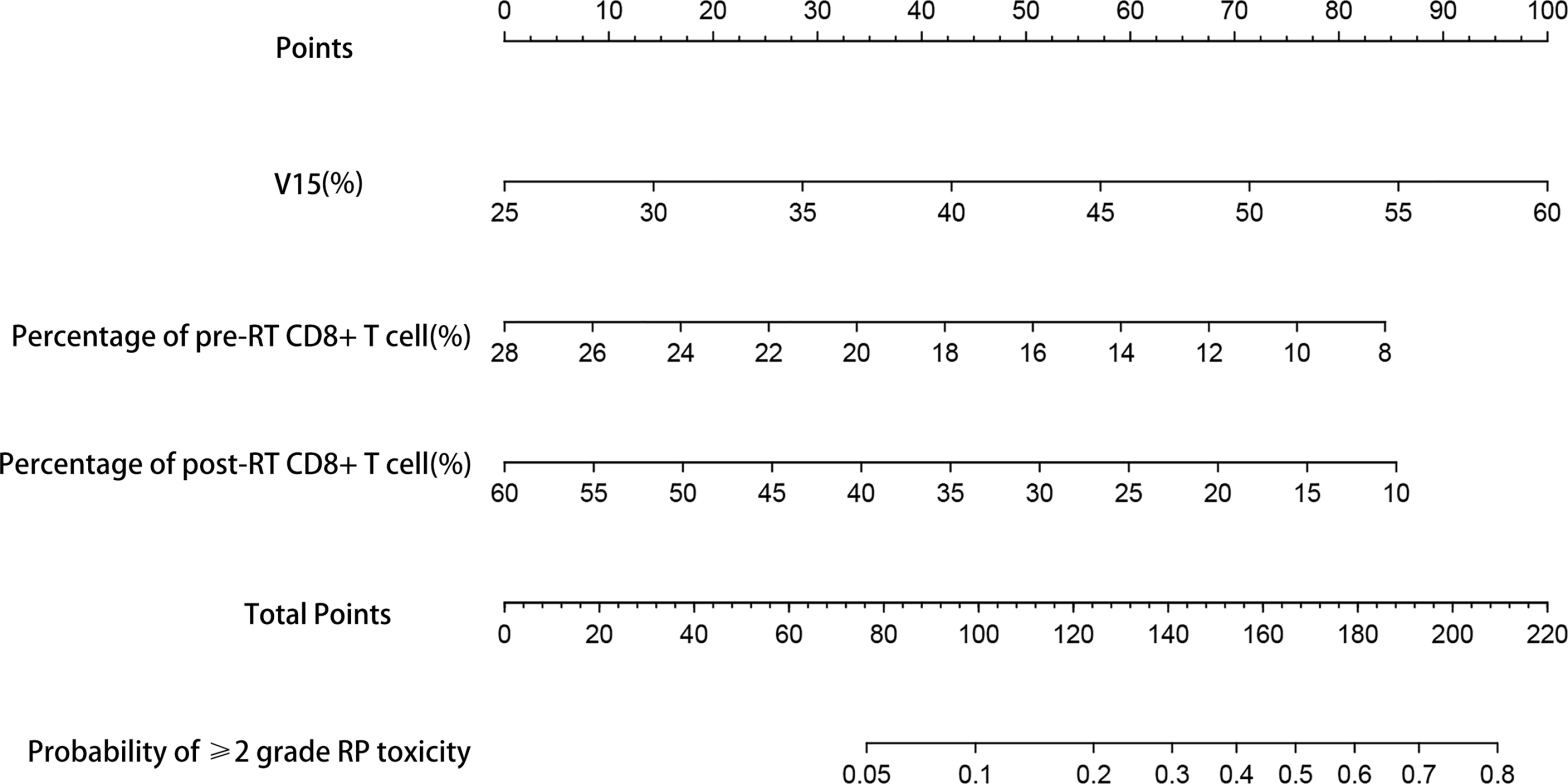
Nomogram of the probability of ≥2 grade RP toxicity in the training cohort. pre-RT, pre-radiotherapy; post-RT, post-radiotherapy.

Based on the results of multivariate analysis, TNM stage, post-RT percentage of CD8+ T cell and lung V15 were used to establish the nomogram to predict the occurrence of RP toxicity (**Figure 1**). And pre- and post-RT percentage of CD8+ T cell, and lung V15 were used to build the nomogram for ≥ grade 2 RP toxicity (**Figure 2**). The length of the line segment represents the contribution to the probability of RP, and the sum of individual scores (points) for each variable is the total score (total points). According to the total points, we can predict the probability of RP and ≥ grade 2 RP toxicity.

**Figure 3:**
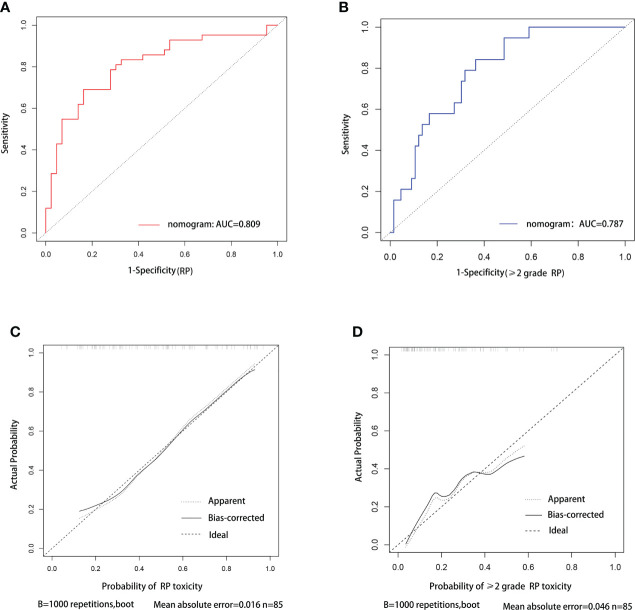
ROC curve and calibration curve analysis for nomograms of RP and ≥2 grade RP toxicity. ROC curve **(A)** and calibration curve **(C)** for nomogram of probability RP toxicity, respectively. ROC curve **(B)** and calibration curve **(D)** for nomogram of probability≥2 grade RP toxicity, respectively.

C-index was used to evaluate the discriminative ability of the models. The closer the C-index is to 1, the better the model can distinguish RP. The C-indexes of the nomogram for the risk of RP toxicity were 0.809 (95% CI: 0.715-0.903) in the training cohort, and 0.718 (95% CI: 0.544-0.892) in the validation cohort, respectively; while the C-indexes for predicting ≥ grade 2 RP were 0.787 (95% CI: 0.685-0.889) in the training cohort, and 0.621 (95% CI: 0.404-0.837) in the validation cohort, respectively. ROC curve analyses were also used to evaluate the predictive ability of the nomograms for RP (**Figure 3A**) and ≥ grade 2 RP (**Figure 3B**), which was consistent with the results of C-index. The nomogram models for predicting RP toxicity and ≥ grade 2 RP toxicity showed satisfactory discriminative ability in either training cohort or validation cohort.

Calibration curves were used to assess the consistency of model predictions with practice. The closer the nomogram curve is to the diagonal line, the more closely the predicted probability matches the actual probability. With respect to the models of RP toxicity (**Figure 3C**) and ≥ grade 2 RP toxicity (**Figure 3D**), the calibration curves all basically agreed with the diagonal.

**Figure 4:**
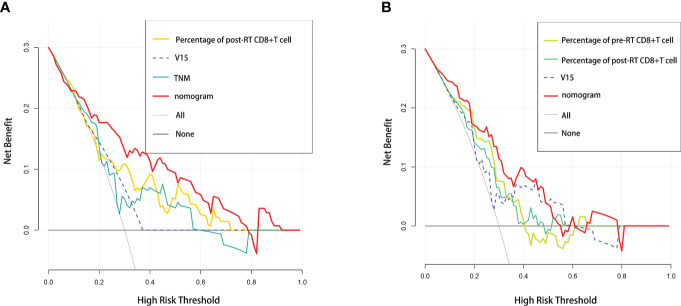
Comparative analysis of net benefit. Decision curve analysis between nomogram and independent predictors in **(A)** RP toxicity model and **(B)** ≥2 grade RP toxicity model, respectively.

In addition, we performed DCA, and found that both of our nomogram models achieved a higher net benefit to predict RP toxicity and ≥ grade 2 RP toxicity (**Figures 4A, B** **)**.

### Comparison between the nomogram and single independent predictor

**Figure 5:**
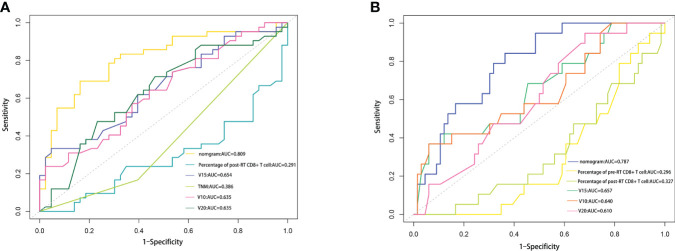
Comparison of the AUC values between nomogram and some influencing factors. **(A)** Comparison of the AUC values among RP nomogram model, independent influencing predictors (percentage of post-RT CD8+ T cell, lung V15 and TNM stage), lung V10 and lung V20. **(B)** Comparison of the AUC values among ≥2 grade RP nomogram model, independent influencing predictors (percentage of pre-RT CD8+T cell, percentage of post-RT CD8+ T cell, and lung V15), lung V10 and lung V20.

To evaluate the predictive optimization of the models, the nomogram was further compared with the single independent predictor identified by multivariate analysis using area under the ROC curve (AUC). Since previous studies have reported that lung V10 and lung V20 are independent predictors of RP (14–17), we also compared our nomogram models with these parameters, respectively. For the prediction of RP toxicity, AUC values of the nomogram, post-RT percentage of CD8+ T cell, TNM stage, lung V10, lung V15 and lung V20 were 0.809, 0.291, 0.386, 0.635, 0.654 and 0.635, respectively (**Figure 5A**). For the prediction of ≥ grade 2 RP, AUC values of the nomogram, pre-RT percentage of CD8+ T cell, post-RT percentage of CD8+ T cell, lung V10, lung V15 and lung V20 were 0.718, 0.296, 0.327, 0.640, 0.657 and 0.610, respectively (**Figure 5B**). Obviously, both of the nomogram models had a higher AUC with respect to predicting RP or ≥ grade 2 RP compared with any single independent predictor.

### Risk stratification based on the nomograms

The total score of RP and ≥ grade 2 RP obtained by summing the specific points from the risk factors was further stratified according to the optimal cutoff of the ROC curve, respectively. In the nomogram for predicting the occurrence of RP toxicity, patients with a total score less than 115.4 were considered low risk, and the opposites were considered high risk. In the nomogram for predicting ≥ grade 2 RP toxicity, patients with a total score of less than 121.2 were considered low risk, and the opposites were considered high risk. We found that the incidence of either RP or ≥ grade 2 RP was higher in the high-risk group than in the low-risk group (**Table 4**), suggesting the nomogram models could help to identify and guide clinical management of patients receiving CRT at high risk of RP toxicity and ≥ grade 2 RP toxicity.

**Table 4:** The incidences of RP in the low- and high- risk groups.

	Without RP	With RP	*p*	RP (grade<2)	RP (grade ≥2)	*p*
Low- risk group	36 (42.4%)	13 (15.3%)	<0.0001	42 (49.4%)	3 (3.5%)	<0.0001
High- risk group	7 (8.2%)	29 (32.6%)		24 (28.2%)	16 (18.8%)	

p-values were calculated using chi-square test or Fisher's exact test.

## Discussion

RP is a common complication of ESCC after CRT. RP may impair the respiratory system, influence radiotherapy efficacy, and lead to pulmonary fibrosis, even death in severe cases (9, 27). Several studies have found that RP is closely related to smoking status, PTV, lung V10, lung V20 and other factors (11, 17, 28). However, there is no ideal markers to predict the risk of RP yet. In this study, we identified that the percentage of CD8+ T cell is closely associated with the risk of RP and ≥ grade 2 RP toxicity.

It is widely accepted that radiotherapy could induce a marked immune response and the release of proinflammatory cytokines by immune cells (29–31). Actually, RP is a kind of immune-mediated hypersensitive pneumonia (32). T lymphocyte subsets play a dominant role in the cellular immune response and may be involved in radiation-induced toxicity (23, 33). Radiotherapy for nasopharyngeal carcinoma can promote CD8+ T cell recruitment by increasing the release of CCL22 (34). PD-1 mediated radiation-induced cardiotoxicity was mainly associated with CD8+ T cell (35). Nakayama et al. (36) revealed that RT could induce the accumulation of CD4+ T cell and CD8+ T cell in lung, which was closely linked to RP. These studies demonstrated that CD8+ T cell may be involved in radiation-induced toxicity including RP. Similarly, in our study, the univariate and multivariate analyses of circulating lymphocyte subsets in patients with ESCC showed that the post-RT percentage of CD8+ T cell was negatively correlated with the occurrence of RP, while pre- and post-RT percentage of CD8+ T cell were negative risk factors of ≥ grade 2 RP toxicity.

CD8+ T cell can be further divided into suppressor T cell and cytotoxic T cell according to their surface markers CD28, and CD8+ CD28- is a surface marker of suppressor T cell (37–39). Since suppressor T cell is one of the major circulating lymphocyte subsets involving in immunosuppression and promote immune tolerance by suppressing effector T cell responses (40–42), we speculated that suppressor T cell may actually contribute to the negative effect of CD8+ T cell on the risk of RP or ≥ grade 2 RP toxicity. Taken together, all these results supported that CD8+ T cell may be involved in the occurrence and development of RP, being an effective marker for RP prediction.

Given the immune regulation role of lymphocyte on RP, we established the nomogram diagnostic models based on circulating lymphocyte subsets. For the prediction of RP, we included TNM stage, post-RT percentage of CD8+ T cell and lung V15 into the nomogram. Notably, in practice, we pay more attention to patients complicated with ≥ grade 2 RP, as these patients are symptomatic, and medical intervention would be needed. How to identify the patients with high risk to develop ≥ grade 2 RP is of great significance. Consequently, we further demonstrated that CD8+ T cell also strongly associated with the risk of ≥ grade 2 RP. And pre- and post-RT percentage of CD8+ T cell, and lung V15 were included for the prediction of ≥ grade 2 RP. Each of our nomogram was proved to be a robust prognostic model by discrimination and calibration analyses. In addition, both models had higher predictive power compared with single independent variables and important risk factors (lung V10 and lung V20). Moreover, DCA results also indicated the models had higher net benefit. According to the cutoff of the model risk score, patients in high-risk group were more likely to develop RP or ≥ grade 2 RP compared with those in low-risk group, suggesting that the models is helpful for risk classification of patients with ESCC receiving CRT.

However, there are several limitations of this study. First, the nomograms were just verified by internal validation, and a multi-center external validation with larger sample size is required. Second, the retrospective design may weaken the reliability for prediction. Last, only esophageal squamous cell carcinoma subtypes were included in this study, and its predictive value for esophageal adenocarcinoma needs further explored.

## Conclusion

In conclusion, TNM stage, post-RT percentage of CD8+ T cell, and lung V15 were the independent predictors of RP toxicity. Pre- and post-RT percentage of CD8+ T cell, and lung V15 were the independent factors of ≥ grade 2 RP toxicity. The nomograms based on circulating lymphocyte subsets can robustly predict RP and ≥ grade 2 RP, guiding clinicians in risk stratification and early intervention.

## Data availability statement

The original contributions presented in the study are included in the article/**Supplementary Material**. Further inquiries can be directed to the corresponding authors.

## Ethics statement

This study was approved by the Ethics Committee of Guangxi Medical University Cancer Hospital.

## Author contributions

All authors listed have made a substantial, direct, and intellectual contribution to the work and approved it for publication.

## Funding

This work was funded by Beijing Xisike Clinical Oncology Research Foundation (Y-2019AZQN-04532), Health Commission of Guangxi Zhuang Autonomous Region (S2021018), China Postdoctoral Science Foundation(2022MD713734), and Guangxi Natural Science Foundation (2018GXNSFAA281057).

## Conflict of interest

The authors declare that the research was conducted in the absence of any commercial or financial relationships that could be construed as a potential conflict of interest.

## Publisher’s note

All claims expressed in this article are solely those of the authors and do not necessarily represent those of their affiliated organizations, or those of the publisher, the editors and the reviewers. Any product that may be evaluated in this article, or claim that may be made by its manufacturer, is not guaranteed or endorsed by the publisher.
