# Supplementary material for: Nomogram based on circulating lymphocyte subsets for predicting radiation pneumonia in esophageal squamous cell carcinoma
Source: Front Immunol. 2022 Aug 29;13:938795. doi: 10.3389/fimmu.2022.938795 (PMC9465326; doi:10.3389/fimmu.2022.938795)
Supplement: Supplementary file 1 [file Table_1.docx]

**Table S1** Correlation of independent variables

| Variables | TNM stage | Percentage of pre-RT CD8+ T cell (%) | Percentage of post-RT CD8+ T cell (%) | Lung V10 (%) | Lung V15 (%) |
| --- | --- | --- | --- | --- | --- |
| TNM stage | 1 | - | - | - | - |
| Percentage of pre-RT CD8+ T cell (%) | 0.091 | 1 | - | - | - |
| Percentage of post-RT CD8+ T cell (%) | 0.078 | 0.154 | 1 | - | - |
| Lung V10 (%) | -0.062 | -0.153 | -0.134 | 1 |  |
| Lung V15 (%) | -0.062 | -0.14 | -0.092 | 0.938***** | 1 |

**Abbreviations:** pre-RT, pre-radiotherapy; post-RT, post-radiotherapy; *, *p*<0.05.
